# Haemophilia in France: Modelisation of the Clinical Pathway for Patients

**DOI:** 10.3390/ijerph19020646

**Published:** 2022-01-06

**Authors:** Karen Beny, Benjamin du Sartz de Vigneulles, Florence Carrouel, Denis Bourgeois, Valérie Gay, Claude Negrier, Claude Dussart

**Affiliations:** 1 Laboratory P2S (Health Systemic Process), UR 4129, Faculty of Medicine Laennec, University Claude Bernard Lyon 1, University of Lyon, 69008 Lyon, France; karen.beny@chu-lyon.fr (K.B.); benjamin.du-sartz-de-vigneulles@etu.univ-lyon1.fr (B.d.S.d.V.); florence.carrouel@univ-lyon1.fr (F.C.); denis.bourgeois@univ-lyon1.fr (D.B.); 2 Central Pharmacy, Hospices Civils de Lyon, CEDEX, 69563 Saint Genis Laval, France; 3 Haemophilia Care Center, Centre Hospitalier Métropole Savoie, 73011 Chambery, France; Valerie.Gay@ch-metropole-savoie.fr; 4 Reference Center on Haemophilia and Other Constitutional Hemorrhagic Diseases, Groupement Hospitalier Est, Hospices Civils de Lyon, 69002 Lyon, France; claude.negrier@chu-lyon.fr

**Keywords:** haemophilia, healthcare management, trajectory, course, modeling

## Abstract

Process-of-care studies participate in improving the efficiency of the care pathway for patient with haemophilia (CPPH) and rationalize the multidisciplinary management of patients. Our objective is to establish a current overview of the different actors involved in the management of patients with haemophilia and to provide an accurate description of the patient trajectory. This is a qualitative exploratory research based on interviews of the principal health professionals of four haemophilia services, between November 2019 and February 2020, in France. Mapping of the CPPH processes within the different institutions and/or services, as well as the rupture zones, were identified. Treatment delivery and biological analyses were carried out exclusively in healthcare institutions. The main liberal health professionals solicited were nurses, physiotherapists and general practitioner. Obstacles and barriers within the specialized service, with other hospital services and external hospital or private services, community health care providers et community environment and individual one was complex and multiples. Our research identified potential concerns that need to be addressed to improve future studies to identify influential elements. Similarly, other qualitative studies will have to be conducted on the perceptions and literacy of patients with haemophilia to develop a global interactive mapping of their trajectories.

## 1. Introduction

Haemophilia A and B (ICD9: 286.0 and 286.1; ICD-10-CM code D66) encompass congenital deficiencies of the intrinsic pathway coagulation factors VIII (FVIII) and IX (FIX), respectively, with a variable risk for bleeding based on the type of haemophilia and the extent of factor deficiency [1]. Approximately 1.1 million of patients are affected by haemophilia worldwide, of whom approximately 418,000 have severe haemophilia [2]. Estimated prevalence is 24.6 cases per 100,000 males for all severities of haemophilia A. It is 9.5 cases per 100,000 males for severe haemophilia A, 5.0 cases per 100,000 males for all severities of haemophilia B, and 1.5 cases per 100,000 males for severe haemophilia B. In 1999, the World Federation of Haemophilia Annual Global Survey identified 78,629 people with haemophilia; this number has since increased to 210,454 in 2018 [3,4]. Since the start of the AGS (Annual Global Survey), there has been a tripling in the number of people identified with all rare bleeding disorders reported. The overview of trends and patterns in haemophilia and its treatment from the World Federation of Haemophilia’s Annual Global Survey Report, between 1999 and 2018, points to an increase in the number of people identified of 267% (210,414 cases in 2018), 295% for Haemophilia A (78,547 cases in 2018)) and 316% for Haemophilia B (78,547 cases) [2]. The global mean (standard deviation) per capita consumption of Factor VIII was 1.57 IU (3.65) in 2002 and 3.91 (1.85) in 2018, while that of Factor IX per capita was 1.57 IU (3.65) in 2002 and 3.91 (1.85) in 2018. America (46%) and Europe (33%) were the highest consumers [3]. The lowest average annual cost per person was reported in Bulgaria (EUR 6660) and the highest in Germany (EUR 194,490) [5]. In France, the median cost of hospitalization for a patient with haemophilia was EUR 30,073 not including the cost of long-acting antihemophilic factors [6]. In 2017, 42,000 people were treated for haemophilia or severe haemostasis disorders, with EUR 565 million allocated to their care. The average annual costs for Haemophilia A management per patient were EUR 72,209.60 [7].

Haemophilia care is complex, often requiring health management beyond the prevention and treatment of bleeding [8]. Over the past twenty years, the therapeutic management of patients with haemophilia has significantly evolved, both with the development of self-treatment and prophylaxis, and the emergence of recombinant drugs and evolution of treatments regarding efficacy and safety. With advances in care, increasing numbers of people with haemophilia achieve near-normal life expectancies [1]. These manifestations are associated with significant direct and indirect costs to individuals, their families, and society that increase in parallel with disease severity [9].

Integration, the coordination and alignment of tasks, has been promoted widely in order to improve the performance of hospitals [10]. Integrated care model is usually delivered by comprehensive haemophilia reference, competence or treatment centre for constitutional haemorrhagic diseases, which provide all components of care including consultation, prophylaxis, technical assistance, medication delivery, and home treatment supervision through geographically co-located and coordinated multidisciplinary teams. Specialized care centres organize care coordination, support technical services, administer finance, provide professional education and participate in the process of collection and reporting of data [8].

The healthcare process of a patient with haemophilia is complex: it is dynamic, variable and multidisciplinary [11]. The phases of the process involve several elements: the specialties of the provider(s), the procedures performed, the diagnoses observed, the therapeutic treatments and the biological analyses [12]. The starting point and end point of the process generally correspond to the beginning and end of the patient’s care. Finally, it corresponds to the overall trajectory of the patient [13]. Management of patients with haemophilia necessarily follows clinical trajectories depending on specific situations [14]. Modelling the clinical pathways of people with haemophilia is part of the process of analysing and providing decision support systems to institutional and field actors involved in the overall and systemic care of patients. Based on the modelling of industrial processes and applied to more diversified and heterogeneous care paths, this is an original and essential step in improving the quality of health care [11]. This approach will make it possible to organize, rationalize and standardize the multidisciplinary management of patients to improve the performance of systems. Process-of-care studies are important to participate in the consideration of improving the efficiency of the haemophilia care pathway as the future of this disease is likely to change with the development of new innovative treatments [15]. Today, research on modelling the care trajectory of patients with haemophilia in specialized reference centres is rare.

This research aims to analyse, model, and provide decision support systems to institutional and field actors involved in the care processes and the global systemic hospital management of haemophilia patients established by the medical system in France. Its objective is to establish a current overview of the different actors encountered by patients with haemophilia throughout their life course, classified by family (hospital, city, etc.), and to identify potential or proven areas of disruption in the life course to improve knowledge of the trajectory of a patient with haemophilia.

## 2. Materials and Methods

### 2.1. Study Design

This is a qualitative exploratory research study. It is based on interviews of the principal actors in the organization of the patient course: health professionals from the hospital teams of four haemophilia services, located in three different regions between March 2019 and February 2020 in France. Using a mixed method, data were collected from the observations and interview manager and staff surveys to collect data on organizational, social, and clinical process integration, at four organizational levels: practice site, physician organization, health system, and outside health systems. Data were used to perform a social network analysis and a structural and functional modelling of the hospital network.

### 2.2. Setting

In France, the haemophilia Care Network is organized into haemophilia treatment centres (HTC). Consultations and care of patients are essentially performed in a haemophilia reference centre (HRC), or a resource and competence centre—constitutional haemorrhagic diseases (CRC-MHC), or a treatment centre for Constitutional haemorrhagic Diseases (CT-MHC). Drugs can be dispensed by hospital pharmacies (retrocession) and since 2021 by city pharmacies.

Four selected Hospital for our study were selected. Each centre specializes in the diagnosis and follow-up of constitutional haemorrhagic and thrombotic diseases:

− Hospices Civils de Lyon (HCL), Groupement Hospitalier Est, is a coordinator site from the HRC and the French network on inherited bleeding disorders (MHEMO) with 3000 consultations per year.
− Assistance Publique des Hôpitaux de Paris (APHP)—Hôpital Bicêtre is a reference centre for haemophilia and other constitutional coagulation protein deficiencies. Nearly 600 patients with haemophilia are followed in this reference centre for rare haemophilia diseases.
− The Lille University Hospital—Lille Regional Haemophilia Treatment Centre (CTL) is one of the five French sites labelled national reference centre for haemophilia and other rare coagulation deficiencies. This centre is composed of several functional units: (i) biological haemostasis; (ii) cardiological haemorrhagic pathology; (iii) haemorrhagic pathology Jeanne de Flandre; (iv) thrombotic pathology; (v) Willebrand UF, vi/Transfusion Safety & Hemovigilance; (vi) immuno-haematology. It should be noted that there is no doctor assigned to each patient, which favours the versatility of medical professionals. It is the doctor on duty who manages, with shared advice.
− The Metropole Savoy Hospital Centre in Chambery (CHC, France) is a CRC-MHC. It should be noted that it is relatively rare to have a haemophilia service in a general hospital, as is the case here. The geography (mountains) partly explains this situation. The number of populations increases by a factor of 100 during the winter vacations (skiing).

### 2.3. Data Collection

A multidisciplinary research team composed of researchers with backgrounds in pharmacy, ergonomics, and public health policy was formed to enrich the free interviews, limit individual interpretations, and strengthen the analyses to meet with several haemophilia services. The planned organization of the day included:

− Interview with the head of the department or his/her representative, who was able to explain the context and history of the organization.
− Visits to the departments concerned: 30 min–1 h.
− Interviews with the different actors of the pathway (collective or individual): doctor, nurse, paediatrician, haematologist, biologist, physiotherapist, pharmacist, etc.: 30 and 45 min.
− Work observations (free time involved) (secretarial/consultation/team meeting, patients, others): observations 1 h or more.
− Debriefing with the Head of Department: 15 min or more depending on availability.

Face-to-face interviews were conducted with physicians, pharmacists, nurses, secretaries, physiotherapists, psychologists, clinical research officers, and nursing assistants. It should be noted that in two cases, the field conditions made it necessary to set up a group interview. A total of 31 people were interviewed. A team meeting was also observed as well as five consultations of patients with haemophilia.

### 2.4. Network Validation

In order to validate, between August 2018 and November 2018, the social network by task was discussed face-to-face with four hospital staff members who also participated in observations and interviews. Some hospital staff members known to perform specific tasks were interviewed via email to obtain details about those tasks. A validation report was prepared to record the comments of the hospital staff.

Two initial pilot interviews were conducted to assess the items for content and conceptual clarity. Results suggested that these items could be used in the specific context of haemophilia care. The study was based on semi-structured interviews with 31 health care professionals: 13 physicians, 3 pharmacists, 1 biologist, 6 registered nurses, 1 physiotherapist, 1 psychologist, 3 clinical research associate, 1 project manager and 2 assistants employed in the French health care system. The interviewees’ responses were analysed using directed content analysis. Maximum variation sampling inside professions were used to achieve diversity in size and performance. The process was set up and coordinated by an ergonomist specialized in the analysis of health trajectories. Individual interviews were conducted by authors (K.B., C.D., B.d.S.d.V.) on behalf of the ergonomist.

An inductive approach was applied using questions based on the existing literature on organizational process and patient trajectories and concerned participants’ experiences and perceptions of the structural organizations they considered to impact their care of patients with haemophilia. The different interviews conducted in these four haemophilia services were followed by discussion meetings within the research team. For each site, the specialist prepared a synthesis, the authors (K.B., C.D., B.d.S.d.V.) meeting to enrich and validate the synopsis. A report of each site visit was written in a collaborative manner. Then, an overall analysis was formulated.

### 2.5. Outcomes

The principal objective was to identify the different actors involved in the management of patients with haemophilia and to determine the similarities between them to provide an accurate description of the patient trajectory.

The secondary objective was to determine any specificities, and their rationale, if applicable.

### 2.6. Sample Size

The number of cases to be included in the study is defined by data saturation and not by a calculation of the number of subjects necessary a priori. Data saturation is achieved when no new concepts emerge from the interviews. In our study, we confirmed data saturation with two additional interviews that did not bring any new concept.

### 2.7. Analysis

The process describes all actors involved in the care of patients with haemophilia. It is described from the point of view of the hospital actors and the intra-site analyses. The weight of each actor is qualitatively weighted, by focusing on the first three sites visited (Lyon/Lille/Paris). The objectives of qualitative analysis were to define concepts, mapping range and nature of process, creating typologies, to identify associations, providing explanations. Framework analysis has been used. Mapping and interpretation involved analysis of key characteristics. This mapping provided a schematic diagram of the event, thus providing guidance to the researcher in their interpretations of the data package.

## 3. Results

**Figure 1:**
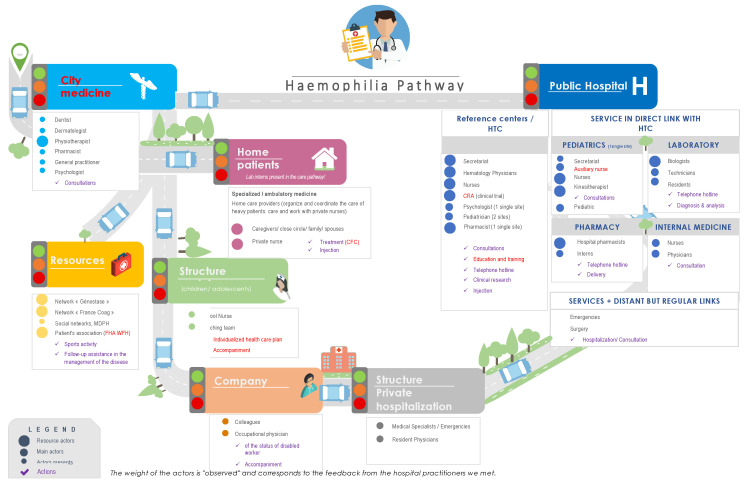
Modelisation of the health course of patients with haemophilia. FHA: French Haemophilia Association; CFC: Clotting Factor Concentrate; CRA: Clinical research associate; HTC: haemophilia treatment centres; mDPH: Departmental homes for disabled people; WFH: World Federation of Haemophilia.

Mapping of the “Care Pathway of Patient with haemophilia” processes within the different institutions and/or services, as well as the rupture zones, were identified (Figure 1).

Following the visits to the four haemophilia hospital services, the following specificities emerge from this representation. For their haemophilia, patients receive support from healthcare professionals, most of whom are hospital-based: medical consultations, treatment delivery and biological analyses are carried out exclusively in healthcare institutions, and in some centres only. The main liberal health professionals solicited were

− Nurses: mainly for treatment injections for patients who do not practice self-injection, or who do not have a caregiver for this practice.
− Physiotherapists: although their role is recognized by caregivers in haemophilia services for managing the consequences of hemarthrosis, they are not very present in the institutions.
− General practitioner is not very present; his or her interactions with the specialized service are very rare.
− Patient’s close family and friends are particularly concerned by the disease, and even involved in its management.

### 3.1. Several Specific Elements Have Emerged

− The estrangement in the relationship between patients and health care teams due to the development of prophylaxis with long-acting treatments.
− Questions about the risk of patients escaping if new treatments are dispensed in pharmacies.
− The haemophilia service sometimes has difficulty getting along with certain other services, particularly emergency rooms, where patients are occasionally poorly recognized and misdirected.
− Poor recognition of women with haemophilia: not only “carriers” but also “affected”: 1/3 of them have factor 8 deficiency. Additionally, 90% of these women are not believed in the emergency room.
− A loss of awareness of the disease on the part of patients, due to the lengthening of the time between treatments or easier administration methods, leading to risky behaviours (such as non-adapted physical activity, non-management of joint pain, etc.).
− The importance of therapeutic education of the patient, and of his entourage, to maintain an awareness of the disease and a good management of it.
− The importance of broad-based support for haemophilia patients, including both physiotherapists and psychologists.
− The link between hospital nurses and private nurses is almost non-existent.
− The link between city doctors and hospital doctors is almost non-existent.
− Adherence to medication was defined as satisfactory in children, average in adolescents, and poorer in adults.

### 3.2. Obstacles and Barriers

#### 3.2.1. Within the Specialized Service

The processes are highly dependent on the organization of the team, the geographical situation, such as the location of centres on several sites, and the spatial location—proximity of the stakeholders. The factors for improving the trajectory are linked to the coordination of the actors because of the possible breakdown in access and the sharing of useful information regarding the patient between internal actors. The levers of multidisciplinary meetings within the CTHs organized around patients, therapeutic indications, and cross-referencing of different types of information are highlighted.

#### 3.2.2. With Other Hospital Services

Historically, relationships are more or less marked and have an impact on the structuring of the service and its organization, for example with the paediatric service at Kremlin Bicêtre. Other services are more in the forefront, such as the analysis laboratory in Lille, where referents participate in team meetings, and the pharmacy in Lyon, where a pharmacist is attached to the service. Relations with other hospital departments are strongly rooted in their collaborative work, depending on the number of staffs, profiles, historical functioning, organization, availability and facilitation of formal or informal exchanges. The aim of these close links is to improve patient care and to optimize it, such as “same-day factor VIII dosage”. In Lille, numerous meetings between manufacturers, pharmacists and doctors for the presentation of treatments have facilitated the work of both, and their collaboration “has simplified referral” and delivery methods based on simulations.

The links with “more distant” services such as emergency services or with centres of complementary and necessary medical expertise (stomatologists) are complex. Difficulties arise from maintaining the skills of people who change and who do not “practice” haemophilia on a regular basis. In Lyon, for example, the CTH participated in the training of emergency physicians, but this action was not sustainable over time (multiple actors/turnover). Protocols were then put in place that necessarily involved the patients (“make us call”). In Chambery, before the implementation of an on-call duty, training was organized every 6 months. Since then, there have been some difficulties maintaining an adequate level of knowledge for emergency residents. In Lille, a briefing is planned every 6 months with the emergency physicians. Similarly, in KB, special orthopaedic consultations for patients with haemophilia are held; in Lille, stomatology consultations are held (once a month) to compensate for the lack of links with the outside world (“some dentists in town refuse to take patients with haemophilia”).

#### 3.2.3. With External Hospital or Private Services

The same necessary relations as with remote intra-hospital services are developed, with the additional difficulty of distance and the organization of processes (who is in charge/thinking about it/checking). Patients are regularly reminded to call the haemophilia service when they go to another institution (travel, surgery or emergency). Patients with haemophilia also sometimes voluntarily hide their disease so as not to extend the length of their stay, for example.

#### 3.2.4. With Community Health Care Providers

The pathway depends on the presence, training and knowledge of those involved in haemophilia and patient care, but also on the patient’s pathway.

Many factors impact on good liaison between these players: distance, lack of training or collaborative tools, involvement in patient follow-up, knowledge of the specificities of haemophilia care. 

The distanced role of the general practitioner in relation to the patient’s course and the number of hospital consultations, for example (depending on the type of haemophilia), making the patient think that their general practitioner is less useful; this would be even more pronounced for women with haemophilia, as highlighted. This raises the question regarding the tools used between the city and hospital actors, via the injection follow-up booklet, the health booklet and the very variable methods of liaison (reports, etc.). For example, in Lille, the service receives two to three calls from general practitioners per year.

There are some exceptions depending on the site, notably in Lille, with physiotherapists (specific list of private physiotherapists—history and proximity link with a Belgian team which has developed many links with physiotherapists). This is not the case with psychologists, with whom attempts at cooperation envisaged in the past have not been satisfying. The avenues envisaged concern a potential mutualization with the rare diseases network.

In Lyon, links are being created between associations of hospital practitioners (nurses, doctors, pharmacists) and associations of community practitioners, while emphasizing the absence of computer links and the digitization of the care record of patient with haemophilia, which is currently underway. For dental, dermatology and colonoscopy care, compliance with protocols is imperative.

The measures envisaged include the mobilization of professional societies and the production and distribution of dedicated documents.

#### 3.2.5. Between the Stakeholders in General Practice

The organization of the care pathway within the general medical practice should be developed in the questioning of patients.

#### 3.2.6. Links between Patients with Haemophilia and Existing Support Structures

The important coordination and weight of the various associations and network organizations around patients and professionals were emphasized. However, although many associations exist, they are not always well identified by patients neither by community practitioners nor other hospital services.

#### 3.2.7. School Care

Difficulties related to a lack of knowledge about haemophilia, difficulties in providing care (school trips, transporting injections, etc.), stigmatization, choice of sports, etc., penalize the children’s trajectory. In Lille, a concern extends to the siblings of patients with haemophilia.

#### 3.2.8. Professional Structures

There are weak links with health professionals, particularly occupational physicians. In Lyon, their importance was underlined, particularly in their necessary involvement in the constitution of files for persons with disabilities. Finally, the impact is weak and may raise the question of keeping patients with haemophilia in employment.

#### 3.2.9. The Immediate Environment—Family

The same level of requirements are necessary for education, and lifelong maintenance of skills is necessary. The understanding of one’s disease, the way one lives with it, beyond the cognitive capacity to understand it, is also an element that influences the course of the disease. The place where one lives and access to care are also parameters that influence the course of treatment. Some patients with haemophilia hide their disease to avoid prolonging a hospital stay when they are in an institution where they are not usually followed up. There is a need to educate the people around them in the same way as the patients, with a similar requirement, and to keep them competent to carry out the injections in the event of difficulty for the patient.

#### 3.2.10. The Impact of Therapeutic Innovations

Therapeutic innovations will have an impact on the treatment pathway, its structure and its actors, and both will have an effect at several levels. First, on the work of practitioners, particularly their medical and professional practices, as well as on dosages and analyses. Second, on hospital-city coordination and possible ambulatory risks linked to the distance of patients from hospitals, as the pathway is considered today. This refers to the structuring of ambulatory care, its coordination, its development, as well as the accessibility of treatments and care in case of delivery of treatments in city pharmacies. The necessary reinforcement of outpatient services and their reorganization so that they can respond to needs would be a real lever in the fight against areas of breakdown. The accessibility of information and the improvement of knowledge about haemophilia, and the resource people to whom one can turn, are issues. Similarly, the impact of the network on the territory may accentuate the disparities in patient care.

Finally, in terms of the impact on patients’ lives and their apprehension of haemophilia, their “feeling” will be different, and their quality of life will be improved. For example, patients affected with a mild case of haemophilia may forget that they have this condition, and joint pain may not be caused by the disease. Other impacts may concern specific cases, such as patients with inhibitors.

## 4. Discussion

To the best of our knowledge, no literature has been published to date on the use and impact of clinical pathways on the quality of care for patients with haemophilia in healthcare settings. The present article provides a qualitative synthesis of the implementation of clinical pathway initiatives for patients with haemophilia in specialized French healthcare services. Its objective was to establish a current picture of the players encountered by patients with haemophilia throughout their life course, classified by family (hospital, city, etc.), and to identify potential or proven areas of disruption in the course. The method was to get a group of people to work on this picture, to have the elements that can vary the pathway of patients with haemophilia and, if fine, to identify all the impacts of therapeutic innovations on the trajectory.

Centres of expertise reference networks for rare diseases, including haemophilia, are fundamental to EU health policy (Article 12) [16]. Standards for the designations of two distinct levels of European health centres and delivery of optimal haemophilia care were introduced in 2008: (i) European Haemophilia Treatment Centres (EHTCs), should normally care for at least 10 people with severe haemophilia, delivering routine local care, and (ii) European Haemophilia Comprehensive Care Centres (EHCCCs), should normally care for at least 40 people with severe haemophilia, delivering multidisciplinary, specialized care and operating as a tertiary centre of reference [17]. Three of the four centres in our study were in the last category. At that time, the 10 guiding principles of haemophilia treatment were defined to provide a benchmark for the treatment of the disease [18]. France has subscribed to these EHCCCs principles. As our study highlights, the trajectories of patients with haemophilia are based on an integrated approach to comprehensive multidisciplinary care. The itinerary described all the stakeholders involved in the management of patients with haemophilia. It is described from the perspective of the hospital professionals and intra–extra-site analyses. This concept of a course is claimed to provide a coherent organization of care and a veritable global service, considering the patient as a whole being, and not just one or several symptoms-diseases to be cared for [19].

The 2018 survey to assess the extent to which the 10 principles of haemophilia care were applied highlighted the principles of haemophilia treatment, and were generally applied across Europe [20]. Our study validates this information for France, whereas for some countries, aspects of national organization of care, centralization, paediatric care, and prophylaxis for adults can be optimized. However, there are still unmet needs in several dimensions of patient care as in general preparation for the ageing haemophilia patient and population psychosocial care [21].

Our research targets care processes, which are certainly restrictive in relation to health or life pathways, but which are the response to health needs on a territory in relation to risk factors and diseases [22]. They involve ambulatory and hospital care, comprising primary care and hospitalization, home hospitalization, follow-up and rehabilitation care, and long-term care units. Contextual analysis should consider the stakeholders who may impact or be affected by the health service, as well as the physical, social, political, and economic environments that may enable or hinder the standardization of the service [23]. Early identification of these elements, including how they relate or interact to one another, is a major first step in the process to develop effective strategies and interventions to strengthen the health service implementation [24].

Whereas until the 1960s, the median life expectancy for a patient with haemophilia was 30 years, the development of effective treatment based on prophylactic replacement of the missing factor, as well as a better knowledge of the disease, has changed the paradigm. Today, the quality of life and life expectancy for individuals with haemophilia is virtually normal [25].

In France, a national pharmacosurveillance system for FVIII and factor IX products administered to haemophiliacs was founded by the public health authorities in 1994 [26]. Renamed FranceCoag in 2003, its competence was also extended to other inherited bleeding disorders [27]. The high observed average prevalence of haemophilia A at birth (23.3 cases per 100,000 male live births between 1991 and 2008) compared with prevalence in other industrialized countries supports the exhaustiveness of this registry [27]. The median age of a cohort of 599 individuals at the time of diagnosis from 1980 to 1994 was 7.7 months. This age differed significantly between the different subgroups: 28.6 months in the mild forms, 9.0 months in the moderate ones and 5.8 months in the severe forms. In the severe forms, a trend towards earlier diagnosis was observed for three consecutive time periods [28].

In 1999,1234 people affected had been registered in 39 haemophilia centres. At the time of admission, 50.2% of them were younger than 15 years of age [26]. In 2005, diagnoses of haemophilia for the 4018 patients included in the 1994–2005 cohort comprised haemophilia A (*n* = 2901), of which 1306 were severe, and haemophilia B (*n* = 605), of which 229 were severe. The median age of patients was between 20 and 26 years according to the type of coagulation disorder, with a median age at diagnosis of 0.7 and 0.8 years for patients with severe haemophilia A and B, 1.7 and 3 years for patients with moderate haemophilia A and B, and 7.6 and 6.7 years for minor forms [29].

The French cohort provided in 2016 data from 9504 patients with hereditary bleeding disorders indicated that 60.5% were affected by haemophilia A and 13.7% by haemophilia B. Median age was 32 years (IQR: 18–50 years). At that time, a serum and plasma sample was available for 27.1% patients [30].

Our research identified four potential concerns that need to be addressed to improve future studies to identify influential elements:

− The importance of the hospital in the current course with the predominance of its actors, who may differ from one site to another, and the close links occasionally difficult to establish with the patient’s life outside the hospital. This is a constant observed in industrialized countries. Procedures, rules for collaboration between EHTCs and EHCCCs must be written, shared and made available to the public [31]. While the implementation of the haemophilia treatment centre model is dependent on the availability of a relevant range of medical and paramedical services, states and territories depending on resources, patient population demographics, centralization and organization of services, most people with haemophilia receive care through specialized centres [32]. For example, in Australia, these centres were established in 1998 to play a leading role within their hospital, city and outlying areas to ensure optimal care and equitable distribution of professional and therapeutic resources [33].
− Links outside the hospital of various categories. The links are occasionally not strong enough to be maintained in the long term, reinforcing the importance and the necessary autonomy of the patient in the management of their illness. The links are occasionally non-existent or created on the basis of opportunities or historical configurations and often dependent persons. Certainly, the World Federation of Haemophilia recommends that patients with haemophilia be managed in a specialized comprehensive care centre [34]. However, sharing best practices with other healthcare professionals external to hospitals allows for better patient-centred approaches, and improved adherence and health outcomes [35]. Today, individuals with haemophilia can look forward to a virtually normal life expectancy and quality of life [25]. In France, 131 national reference centres for rare diseases must organize partnership in the delivery of care and the coordination of the health and social care network. Patient education, information and training of professionals, patients and their families are also among their missions. This raises the question of the extent to which the care of specific diseases or highly specialized interventions are restricted to a limited number of specific centres or health care providers. The treatment of haemophilia is evolving and changed the role of external stakeholders [36]. As a result of progress in disease management, healthcare professionals who are not haemophilia specialists are more likely to encounter people with haemophilia [37]. Nurses, for example, are the first point of contact for people with haemophilia and provide more care to patients and their families than any other specialist on the team [38,39]. They are to place greater emphasis on psychosocial support, therapeutic education, shared decision making, and patient support and advocacy [40].
− Intra-hospital interactions linked to opportunities, specific historical organizations and collaborative choices that are not always possible (limited flexibility) in terms of time, human or financial resources, availability, etc. The mission of French clinical networks of reference centres is to structure and coordinate the activities between the national and regional centres, the technical platforms, or any other structure involved in care for patients. EHTC must have access to multidisciplinary support, locally or in conjunction with EHCCCs as orthopaedics and physiotherapy, dental care, surgery, obstetrics and gynaecology, hepatology, infectious diseases, paediatric, clinical psychology and social worker [17,19,32].
− There is considerable variability despite a willingness to describe a “meta-course”, a “standardized” description that makes it possible to visualize all of the participants who revolve around the patient and the places where patients with haemophilia go. Through daily self-management, patients with haemophilia become experts and partners in their own care. As such, they should be considered as separate essential members of the comprehensive care team [11]. However, main risks of disruption in this process target parameters such as patient typology, geographical accessibility, characteristics of the patient’s disease, non-medical environment and therefore the patient’s environment. The necessary search for balance between standardization of the care action and adaptation to the specificity of each situation, and the precious pursuit of “standardized singularity”: the personalization of standardized steps will contribute to the reduction in health inequalities [31].

Certain points in this research have identified structural weaknesses that provide guidance and decision-making aids for the recommendation or improvement of operational axes. The so-called “specialty” reference centre is the guarantee of quality care for haemophilia, a rare disease that requires a concentration of the necessary targeted financial human resources for a country, France, whose hospital-centred health organization allows for territorial coverage and quality geographic access for patients. This, where the health actors do not have for this disease problems of financing care for the patients, is the state taking in its account the totality of the treatments.

Within these reference centres, the complementarity between the expertise of the professionals and the experience of the patient accumulated through life with his or her health or psychosocial problems, the disease and its repercussions on his or her personal life and that of his or her families must be reinforced: personalizing care, developing and strengthening the patient’s skills in sharing decisions with caregivers, knowing how to pay special attention to the physical and psychosocial health of patients, and continuity of care over time are the principles that guide the patient-centred approach. Improving the training and awareness of a multi-professional and, if necessary, multi-disciplinary team, as well as those of associated professions outside the reference centre, remains a challenge. The priority is to ensure a fluid and secure reception of patients at the right time in a relationship within the emergency services.

The expectation is that all members of the core team should be skilled and experienced in the treatment of bleeding disorders, but this must be accompanied by an awareness that patients with haemophilia do not have the same level of awareness/concern or perfusion capacity. Professionals need to be aware of the physical and psychosocial health of patients. As such, written management protocols are essential to ensure continuity of care, even if clinical staff changes. These protocols should be as personalized as possible, depending on age, venous access, bleeding phenotype, activity, and the availability of clotting factor concentrates. Recommendations for the management of patients with haemophilia are updated and disseminated by the various scientific societies identified.

Continuity of care over time requires awareness and education of other health care providers who are not specialized in haemophilia care [37]. It is a response to the need to improve quality of life, accessibility to treatment and the performance of management for patients treated chronically over the long term. It is in this context, for example, that the members of the Reference Centre for Haemophilia and Other Coagulation Proteins have contributed to the design of various tools made available to the entire community of healthcare professionals, such as the introduction of a dual dispensing circuit for HEMLIBRA^®^ (emicizumab), effective June 2021 [41]. In practice, if a patient wishes to have this drug dispensed in a community pharmacy, an e-learning course called “HEMOPHAR” for the retail pharmacist must be followed. The retail pharmacist must have completed the training modules. Tool sheets will be provided to facilitate the link between the CRC-MHC team members and the pharmacist.

At least, improving recognition of the condition in women is a sensitive issue, especially when the screening is conducted in the office of a community health care provider who is unlikely to consider haemophilia simply because the patient is a woman [42,43]. One-third of female drivers may have factor VIII or IX levels <40% and report bleeding events comparable to men at the same level, with the added risk of menorrhagia and postpartum haemorrhage [44]. Initial training in general medicine, gynaecology, obstetrics, and dentistry, continuing education for external health care providers relayed by academic societies, and awareness-raising among women under the authority of haemophilia patient associations must be reinforced. The management of women with low levels of haemophilia or with haemophilia should be identical to that of men with haemophilia: provision of a haemorrhagic disease card and booklet, regular consultations, correction of haemostasis for trauma or before surgery or dental extractions. Special attention should be given to gynaecological and obstetrical events.

This study presents strengths. The baseline assessment of quality performed in this review attempted to provide a quality minimum as well as identified a large range of items that may influence the course implementation. The methodology of interviews, whether conducted individually or in groups with health professionals, is adequate to assess and characterize a variety of unique influencers. Qualitative methodology may affect the type of barriers/facilitators identified, but it is more likely that the aims of the studies included, their population of interest, and/or the particular service/subject addressed by the study had a stronger impact on the type of obstacles or facilitators identified. This work was deliberately limited to the French context, which allows for directly relevant and applicable results. Thus, focusing only on France appears to be a wise decision and not a limitation because it allowed us to obtain data in a context of particular interest.

A key limitation of this study was the selection bias associated with the identification and recruitment of participants. Volunteers may have been more positive about the expanding role of community professionals.

## 5. Conclusions

This study has been a positive exercise in enabling a greater understanding of the current haemophilia care landscape in France. The identification of barriers and facilitators, including how they relate to or interact with each other in the trajectory care of patients with haemophilia, is a key step for developing suitable strategies and interventions to enhance health service performance. The best knowledge of the care processes of patients among the key stakeholders will help to optimize clinical outcomes while enhancing patient quality of life. The Principles of Haemophilia Care were generally applied in France. Some aspects of national organization of care, interrelationship between key stakeholders—healthcare professionals, patients and their caregivers, managed care professionals—may be improved. Future studies could explore the opinions of a wider range of patients and broaden the scope of research to health and life-course pathways.

## Data Availability

The data presented in this study are available on request from the corresponding author.
